# TAAR8 in the Brain: Implications for Dopaminergic Function, Neurogenesis, and Behavior

**DOI:** 10.3390/biomedicines13061391

**Published:** 2025-06-06

**Authors:** Taisiia S. Shemiakova, Alisa A. Markina, Evgeniya V. Efimova, Ramilya Z. Murtazina, Anna B. Volnova, Aleksandr A. Veshchitskii, Elena I. Leonova, Raul R. Gainetdinov

**Affiliations:** 1 Laboratory of Neuroscience and Molecular Pharmacology, Institute of Translational Biomedicine, Saint-Petersburg State University, 199034 Saint-Petersburg, Russia; t.shemyakova@spbu.ru (T.S.S.); e.v.efimova@spbu.ru (E.V.E.); r.murtazina@spbu.ru (R.Z.M.);; 2 Pavlov Institute of Physiology Russian Academy of Sciences, 199034 Saint-Petersburg, Russia; veshchitskiiaa@infran.ru; 3 Center of Transgenesis and Genome Editing, Institute of Translational Biomedicine, Saint-Petersburg State University, 199034 Saint-Petersburg, Russia; e.leonova@spbu.ru

**Keywords:** TAAR8, TAAR, trace amine, triple knockout, adult neurogenesis, short-term memory, depression, dopamine

## Abstract

**Background/Objectives:** G protein-coupled trace amine-associated receptors (TAARs) belong to a family of biogenic amine-sensing receptors. TAAR1 is the best-investigated receptor of this family, and TAAR1 agonists are already being tested in clinical studies for the treatment of schizophrenia, anxiety, and depression. Meanwhile, other TAARs (TAAR2, TAAR5, TAAR6, TAAR8, and TAAR9 in humans) are mostly known for their olfactory function, sensing innate odors. At the same time, there is growing evidence that these receptors may also be involved in brain function. TAAR8 is the least studied TAAR family member, and currently, there is no data on its function in the mammalian central nervous system. **Methods:** We generated triple knockout (tTAAR8-KO) mice lacking all murine *Taar8* isoforms (*Taar8a*, *Taar8b*, and *Taar8c*) using CRISPR-Cas9 technology. In this study, we performed the first phenotyping of tTAAR8-KO mice for behavioral, electrophysiological, and neurochemical characteristics. **Results:** During the study, we found a number of alterations specific to tTAAR8-KO mice compared to controls. tTAAR8-KO mice demonstrated better short-term memory, more depressive-like behavior, and higher body temperature. Also, we observed changes in the dopaminergic system, brain electrophysiological activity, and adult neurogenic functions in mice lacking *Taar8* isoforms. **Conclusions:** Based on the data obtained, it can be assumed that the physiological TAAR8 role is not limited only to the innate olfactory function, as previously proposed. TAAR8 could be involved in brain function, in particular in dopamine function regulation.

## 1. Introduction

Trace amine-associated receptors (TAARs) belong to a family of G protein-coupled receptors (GPCRs) that initiate a classical cAMP cascade and have been found in vertebrate tissues [1,2,3]. In mammals, there are nine TAAR subfamilies with different numbers of functional genes due to frequent pseudogenization events [4]. In humans, TAAR1, TAAR2, TAAR5, TAAR6, TAAR8, and TAAR9 are functional receptors, while TAAR3, TAAR4, and TAAR7 are pseudogenes. Most mammalian TAARs, except for TAAR1, are expressed in the olfactory epithelium and olfactory bulb and are involved in the perception of innate stimuli, such as pheromones, the presence of predators, and spoiled food [5]. TAAR1, the most studied member of the family, is widely represented in limbic and monoamine brain systems, playing a role in the modulation of monoamine and glutamate signals [5]. Today, TAAR1 is being actively studied in the context of mental and neuropsychiatric diseases, and TAAR1 agonists are being considered therapeutic agents for schizophrenia, anxiety, and depression treatment [6,7,8,9]. Other TAARs (TAAR2-TAAR9), for a long time, remained without the due attention of researchers and were considered exclusively olfactory [10]. However, with emerging transcriptomic data and transgenic animal experiments, the role of the remaining TAARs in the mammalian brain is becoming compelling. Currently, it is known that TAAR2 and TAAR5 are expressed in the mammalian limbic system and are involved in the regulation of dopamine (DA) and serotonin (5-hydroxytryptamine (5-HT)) functions, emotional behaviors, and neurogenesis [11,12]. These data indicate that these TAARs may become attractive psychopharmacological targets, along with TAAR1.

Knowledge about TAAR8 and its functions remains limited. While only one isoform of *TAAR8* is found in humans, three genes encoding three isoforms of this receptor were identified in mice: *Taar8a*, *Taar8b*, and *Taar8c*. Human TAAR8 is expressed in the olfactory epithelium; a low mRNA content was also found in the hippocampus, amygdala, and neocortex [3,4,13,14,15,16]. Computer modeling has enabled the detection of a potential binding pocket for diamines such as putrescine and cadaverine, but their role as selective ligands has not yet been reliably confirmed [17]. A specific component of the urine of carnivorous and non-carnivorous animals activates the TAAR8c receptor in rats [18]. Several studies have demonstrated rat and mouse TAAR8 isoforms’ affinity to 1-methylpiperidine, N,N-dimethylcyclohexylamine, piperidine, and N,N-dimethyloctylamine in submicromolar concentrations [19]. Currently, no studies exist describing the involvement of TAAR8 in animal behavior, as well as its role in the central nervous system. Since TAAR8 remains poorly characterized and the correspondence between mouse isoforms and the single human isoform is unknown, we created a knockout animal model lacking all three isoforms of the *Taar8* murine gene (triple TAAR8abc–KO mice and tTAAR8-KO) by CRISPR-Cas9 technology and conducted a comprehensive study of their behavior, neurochemistry, histology, and electrical activity of the brain to reveal TAAR8’s function.

## 2. Materials and Methods

All animal studies conducted in the field of animal research at Saint-Petersburg State University were approved by the Saint-Petersburg State University Bioethics Committee (Approval № 131-03-5, 11 October 2022; № 131-03-9, 25 September 2023). The procedures strictly adhered to the guidelines outlined in the Guide for the Care and Use of Laboratory Animals.

### 2.1. Generation of TAAR8-KO Mice by CRISPR-Cas9 Technology

Surgical interventions were performed under general anesthesia using intraperitoneal injections of TZ (30 mg/kg; Zoletil, Virbac Lab, Carros, France) and xylazine (6 mg/kg; Interchemie Werken de Adelaar, Castenray, The Netherlands).

The selection of spacer sequence for single guide RNA (sgRNA) constructs was carried out using the CHOPCHOP program (http://chopchop.cbu.uib.no/; accessed on 1 September 2022). In vitro transcription was performed with T7 RNA polymerase (Invitrogen, Waltham, MA, USA), and the sgRNA sequence is provided in Table 1. RNA isolation and purification were accomplished using the MEGAclear™ Transcription Clean-Up kit (Invitrogen, USA).

To acquire fertilized eggs, C57Black/6 female mice, aged between 4 and 5 weeks, were used. These mice had previously undergone superovulation, which involved the injection of 7U of foal mare serum gonadotropin and 7U of chorionic gonadotropin into their peritoneal cavity, and then mated overnight with C57Black/6 males. Zygotes were collected from the oviducts of C57Black/6 female mice with copulation plugs. Following that, zygotes were transferred into the M2 embryo culture medium (Sigma, St. Louis, MO, USA) for the process of microinjection. A combination of Cas9 mRNA (20 ng/μL) (Invitrogen, USA) and sgRNA (40 ng/μL) was prepared and then injected into the cytoplasm of the zygotes using a FemtoJet microinjector (Eppendorf, Hamburg, Germany) with constant flow settings. The precise procedure for cytoplasmic microinjection has been comprehensively explained by Doe et al. [20]. The microinjected zygotes were cultured in a KSOM embryo culture medium (Sigma, USA) at 37 °C under 5% CO_2_ for approximately one hour. Subsequently, successfully microinjected embryos (around 8–12) were transferred into one oviduct of pseudopregnant CD1 mouse females with copulation plugs. To induce pseudopregnancy, mature recipient CD1 females were mated with vasectomized CD1 male mice the day before surgery.

The pups (F0) obtained were genotyped by PCR and confirmed by DNA sequencing analysis. Genomic DNA was extracted from the mice’s ears and analyzed by PCR using primers listed in Table 1. Primers for amplification and sequencing were selected using the PerlPrimer program [21]. Analysis of sequencing chromatograms was conducted using the Synthego computer program (https://www.synthego.com/; accessed on 1 September 2022).

### 2.2. TAAR8a mRNA Expression Analysis by RT-qPCR

To analyze the expression of the rat *Taar8a* gene, we used a group of Sprague Dawley male rats (n = 4, age 3–7 months). The following brain structures were taken: the olfactory bulb, medial prefrontal cortex, midbrain (the substantia nigra and the ventral tegmental area, separately), nucleus accumbens, ventral hippocampus, amygdala, hypothalamus, and medulla oblongata. Tissues were dissected on ice, immediately placed in liquid nitrogen, and stored at −80 °C until processing. RNA isolation was performed using TRI Reagent (MRC, New York, NY, USA). The RNA pellet was resuspended in RNase-free water and kept at −80 °C until used. For cDNA synthesis, 0.5–1 μg of RNA was used with Revertaid Reverse Transcriptase (Thermo Scientific, Waltham, MA, USA) in a total volume of 30 µL. A Turbo DNA-free kit (Thermo Scientific, USA) was used to digest any remaining genomic DNA. Each sample was exposed to the same treatment as a control for the successful removal of genomic DNA, except that the reverse transcriptase was not added. For qPCR, 1 μL of undiluted cDNA (a technical triplicate) was used as a template with a qPCRmix-HS SYBR mix (Evrogen, Moscow, Russia) with primers for the *rTaar8a* gene (forward primer 5′-GGACAGAAATCCCTCAAGCATC-3′; reverse primer 5′-CGGAGCCCTGGCGAATAG-3′ [22]) for a qPCR reaction in a CFX96 machine (Bio-Rad, Hercules, CA, USA). The rat Hprt gene was included as an internal control [23]. The amplification specificity was confirmed by melting-curve analysis (from 55 to 95 °C), 2% agarose gel electrophoresis, and sequencing of the PCR product. The normalization and analysis of qPCR data were performed using the ΔΔCt method [24].

### 2.3. Animals

All experiments used 3–6-month-old male tTAAR8-KO and control C57Black/6 mice. Mice were housed in groups of three to five per cage and maintained under standard laboratory conditions (12 h light/dark cycle, 21 ± 1 °C, and 40–70% humidity) with food and water provided ad libitum. All experiments were conducted during the light phase, and the same types of experiments were conducted at the same short period of the day.

### 2.4. Behavioral Tests

Behavioral tests were performed sequentially as follows: circular open field, open field with two objects, open field with novel object recognition, marble burying test, T-maze test, light–dark transition test, elevated plus maze test, grip strength test, stress-induced hyperthermia test, hot plate, and forced swimming test. A total of 12 tTAAR8-KO males and 12 control males, 4–6 months old, were used in the experiments. One hour before the behavioral experiments, mice were habituated to an experimental room. During experiments, video recording of animal behavior was conducted. The analysis of behavioral parameters was carried out using EthoVision software version 15 (Noldus, Wageningen, The Netherlands).

#### 2.4.1. Open Field Test

For testing, a circular arena made of gray plastic was used, which was 63 cm in diameter, with 40 cm high walls, and equipped with 13 holes (diameter 1.6 cm). Animals were placed in the center of a brightly lit empty arena, where they could move freely for 5 min. The total distance traveled, cumulative duration in the central zone, number and total time of grooming episodes, number of rearings, and number of hole explorations were registered during the tests.

#### 2.4.2. Open Field with Novel Objects

First, two identical objects were placed in an open field arena at the same distance from the center and the walls of the arena. Each mouse was placed in the center of the arena for 5 min. During the test, the frequency and total time of interactions with objects were assessed.

The next day, the reaction to a novel object was assessed. In the arena, one of the objects remained the same as it was on the previous day (“familiar”), and the second object was changed to a different shape (“novel”).

Each animal was placed in the center of the arena. It could move and interact with objects for 5 min. The frequency and total time of mouse interactions with “familiar” and “novel” objects were assessed.

#### 2.4.3. Marble Burying

Transparent plexiglass cages (size: 27 × 16 × 10 cm) filled with 5 cm of wooden bedding were used. Twelve balls of solid, dense material, 1 cm in diameter, were placed equidistant on the surface of the bedding. Each mouse was placed in a cage with balls for 30 min. After 30 min, the number of buried balls was counted. The ball was considered buried if at least 2/3 of its volume was covered with bedding.

#### 2.4.4. T-Maze Test

The apparatus was a maze consisting of a central (starting) arm (38 × 10 cm) and two side arms (28 × 10 cm each) with 20 cm high walls. One or both side arms could be either open or closed with a wall. During the experiment, each mouse was placed in the maze twice for 5 min with a 60 min interval. During the first trial, one of the side arms was closed. The mouse was habituated to the open arm (hereinafter referred to as “familiar”). During the second trial, both side arms were opened. During the test, the time spent in novel and familiar arms of the maze was evaluated.

#### 2.4.5. Light–Dark Transition Test

The apparatus consisted of two compartments (20 × 20 cm each): a highly illuminated light zone with an open top and a dark zone with a closed top, with a passage between the light and dark zones sized 3.5 × 3.5 cm. The mouse was placed in a light area. Within 3 min, the animal could move freely in the apparatus. Latency of entering the dark zone, time spent in the light zone, and number of nose pokes into the light chamber from the dark zone were evaluated.

#### 2.4.6. Elevated Plus Maze Test

The apparatus was a plus-shaped platform located at a height of 40 cm above the floor level. The platform consisted of two open arms (30 × 5 cm) located opposite each other and two closed arms (30 × 5 × 15 cm). Each mouse was placed at the intersection of open and closed arms with its nose towards one of the open arms and was allowed to explore the maze for 5 min. The following parameters were measured: total time spent in the open arms, duration of grooming, number of rearings, and number of head dips in the open arms.

#### 2.4.7. Grip Strength Measurement

The test evaluated the strength of the forelimbs. The mouse was lifted by the tail and moved towards the bar of the power meter (BIO-GS4, BIOSEB, Vitrolles, France) until it established a firm grip with both forepaws. When the mouse held the bar, it was pulled away by the tail in a gentle manner until its grasp was broken. The force of the animal grip was evaluated.

#### 2.4.8. Stress-Induced Hyperthermia Test

The test analyzed the dynamics of changes in body temperature under restraining stress. Restrained mice received three sequential rectal temperature measurements with a digital rodent thermometer (BIO-TK8851, BIOSEB, France). The first measurement showed the basal body temperature. The second measurement was carried out 15 min later, and the third measurement was carried out 30 min after the first measurement. The core body temperature obtained by successive measurements was assessed.

#### 2.4.9. Hot Plate Test

Pain sensitivity under thermal exposure was analyzed. The test was carried out using the Hot/Cold Plate apparatus (BIO-CHP, BIOSEB, France). Each mouse was placed on a metal plate measuring 16 × 16 cm, preheated to a temperature of 52 °C. After the first painful reactions (pulling paws away from the surface and shaking or licking the paws), the surface heating was turned off, and the animal was removed from the plate. The latency of the first pain reaction was evaluated.

#### 2.4.10. Forced Swimming Test

The forced swim test was conducted to evaluate depressive-like behavior. A tall glass cylinder with a height of 21.5 cm and a diameter of 10 cm was used, filled with water at a temperature of 21–22 °C. The water level (approximately 18 cm) was sufficient for the mouse not to touch the bottom with its tail. Mice were placed individually in cylinders for 10 min. At the end of the test, each animal was removed from the water, dried with a towel, and then placed in a dry and warm place. After each animal, the water in the cylinder was changed. The latency and duration of immobilization were evaluated.

#### 2.4.11. Prepulse Inhibition Test

The prepulse inhibition test was used to measure the effectiveness of sensorimotor gating. Experiments were conducted on 5 male mice tTAAR8-KO and 5 control males aged 4 months. The test was conducted using a custom apparatus for acoustic startle response constructed in our laboratory previously, as described in [25]. Each mouse was placed in a small transparent cylinder (8 × 3.5 cm) mounted on a piezoelectric accelerometer to register motor reactions. Speakers were placed on the sides of the cylinder. In the experiment, each animal was habituated to “white noise” with an intensity of 74 dB for 10 min. Then, against the background of white noise, 20 sound stimuli with an intensity of 100 dB and a duration of 50 ms (“pulse”) were presented, causing a startle response. Next, 20 paired combinations of sound stimuli were presented, consisting of a quieter prepulse (intensity 78 dB) and a subsequent pulse. The interval between the prepulse and pulse stimuli was 100 ms. The interval between the presentation of stimuli or paired stimuli varied from 10 to 14 s. The value of prepulse inhibition was calculated using the formula PPI = (1 − amplitude of paired stimuli response/amplitude of pulse response) × 100. The signals were recorded using Spike2 software version 11 (Cambridge Electronic Design, Cambridge, UK) and an analog-to-digital converter CED Power1401 (Cambridge Electronic Design, UK), synchronized with a system for supplying sound stimuli and video recording of the animal’s motor reactions.

### 2.5. Electrophysiological Recordings

#### 2.5.1. Operating Procedure

Electrophysiological studies were conducted on male mice aged 4 months: tTAAR8-KO (n = 6) and control group (n = 6). For electrocorticogram (ECoG) recording, epidural electrodes (0.5 mm in diameter; 1 mm in length; steel) were used. For local field potential (LFP) recording, intracerebral electrodes (50 mcm in diameter; from 1.2 mm to 3.2 mm in length) made of tungsten wire in polymer insulation were used. Implantation of electrodes in each animal was performed under general gas anesthesia using isoflurane. Four electrodes were implanted in each animal: an epidural reference electrode 2 mm in front of the bregma and 1 mm lateral to the midline on the left; an epidural electrode in the area of the primary motor cortex on the right (M1), 1 mm anterior to the bregma and 1 mm lateral to the midline; an epidural electrode in the area of the primary somatosensory cortex on the right (S1), 1.5 mm posterior to the bregma and 3 mm laterally to the midline; and an intracerebral electrode in the dorsal striatum on the left (Str), 3.2 mm in length, 0.5 mm posterior to the bregma, and 2.5 mm laterally to the midline. The electrodes were installed using a stereotactic micromanipulator. The implanted electrodes were fixed on the surface of the skull using dental cement.

#### 2.5.2. Electrocorticogram and Local Field Potentials Recording

Electrical activity was recorded in an experimental setup that included an isolated grounded camera, an amplifier (gain factor ×1000), and an analog-to-digital converter CED with a pre-installed program Spike2 (Cambridge Electronic Design, UK). The mouse was connected to the apparatus and placed in a plexiglass box measuring 20 × 20 × 25 cm, where it could move freely. The electrical activity of brain structures was recorded for 30 min. Registration and analysis of electrical activity were performed in the Spike2 program (Cambridge Electronic Design, UK). The digitization frequency of the signal was 1250 Hz (measurements per channel per second). The analysis of the obtained data was carried out using the Fourier transform, investigating the relationship between the power and frequency of the signal (LFP recorded in the dorsal striatum and ECoG in the primary motor cortex). Analysis was performed on a set of three-second fragments of the recording corresponding to the awake/research behavior of the animal. In statistical analysis, the 0–1 Hz range was excluded due to the large number of artifacts.

### 2.6. High-Performance Liquid Chromatography Measurements of the Tissue Monoamine and Metabolite Content

The measurements were performed on 8 tTAAR8-KO male and 8 control male mice aged 6 months. The quantitative content of norepinephrine (NE), DA, 3,4-dihydroxyphenylacetic acid (DOPAC), homovanillic acid (HVA), 5-HT, and 5-hydroxyindoleacetic acid (5-HIAA) in the frontal cortex and striatum was analyzed by reverse-phase high-performance liquid chromatography with electrical detection, as described previously [26]. Dissection of the cortex and striatum was carried out on a cooled surface; the structures were frozen in liquid nitrogen and then stored at a temperature of −80 °C. Before the measurement, the samples were homogenized in 0.1 M HClO4 with internal standard 3,4-dihydroxybenzylamine and centrifuged (10 min, +4 °C, 14,000× *g*), and the supernatant was filtered using centrifuge filter units (polyvinylidene fluoride membrane; pore size, 0.22 μm, Millipore, Burlington, MA, USA). An Eicom HTEC-500 device (Tokyo, Japan) was used for analysis; separation was performed on a CA-50DS column (150 × 2.1 mm, Eicom, Japan). Detection was carried out on an analytical cell with a graphite electrode WE-3G (Eicom, Japan) at a potential of +650 mV. The composition of the mobile phase was as follows: 100 mM sodium phosphate buffer, 0.17 mM EDTA, 1.8 mM octyl sulfate, 19% methanol, and pH = 4.56. All the obtained peaks of values were normalized to standard 3,4-dihydroxybenzylamine. The concentration of the analyzed substances was calculated in ng/mg of tissue.

The overall intensity of DA metabolism was estimated by the ratio of metabolite concentrations summed to the DA level (DOPAC + HVA)/DA). The HVA/DA and DOPAC/DA ratios serve as indicators of the DA turnover.

### 2.7. Immunohistochemistry

#### 2.7.1. Perfusion and Primary Tissue Processing

Under deep anesthesia (a mixture of 200 mg/kg Zoletil (Virbac Lab, France) and 16 mg/kg Xylazine (Interchemie Werken de Adelaar, The Netherlands), all animals were transcardially perfused with 0.9% NaCl (100 mL), followed by 4% paraformaldehyde (100 mL) in 0.1 M PBS at pH 7.4. After perfusion, the brains were removed, post-fixed in the fixative for 12 h, and passed through increasing concentration (20–30%) sucrose solutions for cryoprotection. Then, 50 μm frontal free-floating sections were prepared on a cryotome (Reichert, Vienna, Austria).

#### 2.7.2. Tyrosine Hydroxylase Immunofluorescence Staining

For tyrosine hydroxylase (TH) immunofluorescence, we used 5-month-old male adult mice (n = 4) for both the tTAAR8-KO and control groups. To assess TH expression in the mouse brain neurons, an indirect immunofluorescence method was used. Coronal sections were collected at the ventral tegmental area (VTA) and pars compacta of substantia nigra (SNpc) approximately 3.2 mm behind the bregma. The sections were processed in citrate buffer (pH 6.0) for antigen retrieval and blocked with 5% normal goat serum (Elabscience, Houston, TX, USA) for 1.5 h before incubation with polyclonal rabbit anti-TH antibodies (Santa Cruz Biotechnology, Cat # sc-14007, RRID: AB_671397, Santa Cruz, CA, USA, dilution 1:500) at 4 °C, overnight. They were then incubated with Alexa Fluor 488 goat anti-rabbit IgG (Invitrogen, Cat # A-11008, RRID: AB_143165, USA, dilution 1:500) at 37 °C for 2 h and mounted in aqueous medium Fluoroshield with DAPI (Sigma-Aldrich, Cat # F6057, USA).

#### 2.7.3. Doublecortin Immunofluorescence Staining

For doublecortin (DCX) immunofluorescence, we used 5-month-old male adult mice (n = 4) for both the tTAAR8-KO and control groups. For analysis, we took sections approximately from 0.2 to 0.14 mm before the bregma for the subventricular zone (SVZ) and from 1.7 to 2.2 mm behind the bregma for the subgranular zone (SGZ) of the dentate gyrus. The sections were washed, permeabilized, and blocked. To detect neuronal precursors, we used polyclonal rabbit anti-DCX antibodies (Abcam, Cat # ab18723, UK, dilution 1:1000). Incubation in primary antibodies was performed overnight at +4 °C; Cy™3 AffiniPure donkey anti-rabbit IgG (Jackson ImmunoResearch, Cat # 711-165-152, RRID: AB_2307443, Cambridge, UK, dilution 1:800) was used as secondary antibodies at 37 °C for 2 h and mounted in aqueous medium Fluoroshield with DAPI (Sigma-Aldrich, Cat # F6057, USA).

#### 2.7.4. Image Processing

After immunostaining, sections were imaged on a fluorescence microscope (Leica DM4000, Wetzlar, Germany, with a 10× and 20× objective for TH and DCX, respectively) with a built-in black-and-white high-sensitivity digital CCD camera with a resolution of 1.3 MP. The series of images was merged into the resulting one in Adobe Photoshop. Immunopositive neurons and the distribution area (for the SVZ and SGZ) were manually counted using Fiji ImageJ software version: 2.9.0/1.53t (U. S. National Institutes of Health, Bethesda, MD, USA). Finally, we analyzed the average number of TH^+^ neurons and the distribution density of DCX^+^ neurons.

## 3. Results

### 3.1. Generation of tTAAR8-KO Mice by CRISPR-Cas9 Technology

The *Taar8a*, *Taar8b*, and *Taar8c* genes exhibit high nucleotide sequence homology (Supplementary Figure S1). This similarity presented a significant challenge for designing isoform-specific CRISPR sgRNA for each gene and analyzing the resulting mutation after generating a knockout mouse model. To overcome this hurdle, we designed a unique sgRNA capable of targeting each of the respective genes. This approach allowed us to successfully create a new knockout mouse line with all three *Taar8a*, *Taar8b*, and *Taar8c* genes inactivated. In order to design appropriate primers for determining which *Taar8* isoform was knocked out in newborn pups through PCR analysis, 5′ and 3′ exon sequences using the ClustalW program (http://www.genome.jp/tools-bin/clustalw; accessed on 1 September 2022)were compared. The alignment results reveal a high degree of homology in the 5′-end sequence. Consequently, we selected reverse primers from the 3′-end region for the DNA analysis (see Supplementary Figure S2).

Analysis of the F0 offspring identified frameshift mutations occurring in every gene. We selected the F0 mouse with a deletion of 5 base pairs (bps) in the *Taar8a* gene and the deletion of 4 bps in the *Taar8b* gene, and 29 bp deletion was detected in the *Taar8c* gene. This mouse was crossed with a wild-type mouse to obtain heterozygous F1 mice, and after the subsequent crossing of F1 mice, we selected tTAAR8-KO mice bearing three homozygous mutations in *Taar8a* (−5 bps), *Taar8b* (−4 bps), and *Taar8c* (−29 bps).

### 3.2. Taar8a Expression Pattern in the Brain

**Figure 1:**
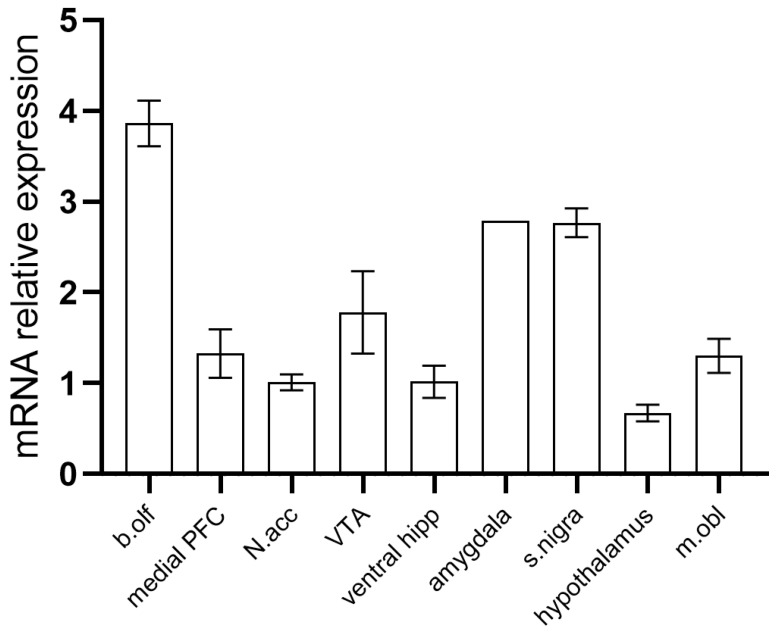
*Taar8a* expression in the rat brain. RT-qPCR was performed on total RNA isolated from structures obtained from adult male rats (n = 4) using oligonucleotide primers for the *Taar8a* gene. Nucleotide sequencing confirmed the specificity of the PCR product. Data are presented as mean ± SEM. b.olf, olfactory bulb; PFC, prefrontal cortex; N.acc, nucleus accumbens; VTA, ventral tegmental area; hipp, hippocampus; s.nigra, substantia nigra; m.obl, medulla oblongata.

While previous studies have characterized *Taar8* mRNA in mouse brains [1,27,28], we examined *Taar8a* expression in rat brains using RT-qPCR. As most *Taar* genes consist of one exon, excluding false-positive results produced by genomic DNA was crucial. Thus, we included no RT control for all samples. The specificity of the PCR product was confirmed by gel electrophoresis and sequencing. Housekeeping transcripts (Hprt) were identified in all probes analyzed with a Ct average of 21.8, and *Taar8a* was lower at a Ct average of 29.6. The highest *Taar8a* expression was found in the olfactory bulb. Interestingly, *Taar8a* appeared to be expressed in all the brain structures studied (Figure 1).

### 3.3. Behavioral Profile

tTAAR8-KO mice showed no general health, development, growth, or body weight alterations. In addition, the genotype and sex distribution were normal. We assessed the behavioral profile of tTAAR8-KO mice using a comprehensive test battery evaluating locomotor and exploratory activity, anxiety levels, compulsive behavior, stereotypical behavior, pain sensitivity, etc.

The analysis of parameters in the open field test, marble burying test, light–dark transition test, elevated plus maze test, grip strength test, and hot plate test failed to reveal statistically significant differences between mutant and control mice.

**Figure 2:**
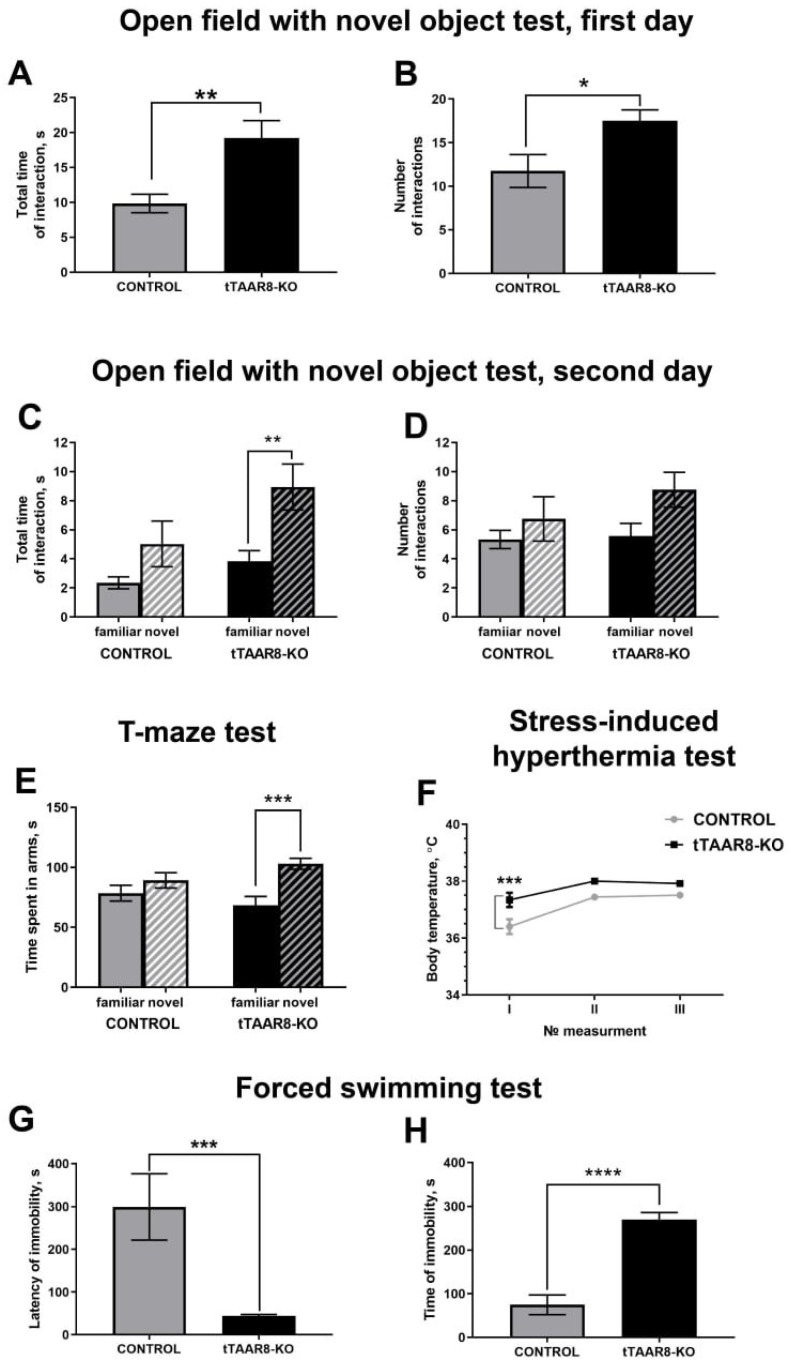
Behavioral profile of tTAAR8-KO mice. (**A**–**D**) Open field with novel object test: (**A**) total time and (**B**) number of interactions with two objects during the first day; (**C**) total time and (**D**) number of interactions with the novel object compared to the familiar one during the second day of the test. (**E**) Time spent in the novel arm compared to the familiar one in the T-maze test. * *p* < 0.05, ** *p* < 0.01, *** *p* < 0.001, Mann–Whitney test. (**F**) Stress-induced hyperthermia test. I—the first measurement; II—temperature measured 15 min after the first measurement; III—temperature measured 30 min after the first measurement. *** *p* < 0.001, 2-way ANOVA. (**G**,**H**) Forced swimming test: (**G**) latency of immobilization and (**H**) time of immobilization. *** *p* < 0.001, **** *p* < 0.0001, Mann–Whitney test. All results are shown as mean ± SEM.

In the novel object test, the cumulative time of interaction with the objects was higher in tTAAR8-KO compared to the control group on the first day of the test (19.22 ± 2.51 s, 9.86 ± 1.32 s, *p* < 0.01) (Figure 2A). Also, tTAAR8-KO mice interacted with the objects significantly more frequently compared to control mice (17.5 ± 1.25, 11.75 ± 1.9, *p* < 0.05) (Figure 2A). There was no difference in lateral preference for the objects in both groups. On the second day of the test, when mice had one novel and one familiar object, tTAAR8-KO mice interacted with a novel object significantly longer than with a familiar one (8.94 ± 1.58 s, 3.83 ± 0.73 s, *p* < 0.01) (Figure 2C), with no difference in the number of interactions (Figure 2D). In the control group, no differences were found in the time (*p* = 0.247) and frequency (*p* = 0.808) of interaction between a familiar and novel object.

In the T-maze test, an assessment of the time spent by the animal in familiar and novel arms was carried out. A comparative analysis of the duration of time spent in arms shows that tTAAR8-KO mice spend significantly more time in a novel arm than in a familiar one (103 ± 4.53 s, 68.34 ± 7.42 s, *p* < 0.001), whereas no difference in the control group was found (*p* = 0.256) (Figure 2E).

The stress-induced hyperthermia test was conducted to assess the intensity of vegetative reactions (body temperature) under restraining stress. In the experiment, the body temperature of the mouse was measured three times. At the first measurement, before the stress reaction, tTAAR8-KO mice had significantly higher body temperature compared to mice of the control group (37.34 °C ± 0.251, 36.4 °C ± 0.264, *p* < 0.001). A total of 15 and 30 min after the first measurement, the differences in body temperature between the groups of mice were not statistically significant (*p* = 0.074 and *p* = 0.268) (Figure 2F).

The forced swimming test was used to observe depressive-like behavior in response to acute, inescapable stress. Latency to the first and total time spent by the animal in an immobile state were measured. It was found that tTAAR8-KO mice become immobile faster than control group mice (44.25 ± 3.71 s, 299.1 ± 77.31 s, *p* < 0.001) (Figure 2G). At the same time, the total duration of immobility in animals of the tTAAR8-KO group is significantly higher than that in the control group (270 ± 16.5 s, 74.67 ± 22.41 s, *p* < 0.0001) (Figure 2H).

### 3.4. Evaluation of the Effectiveness of Sensorimotor Gating

**Figure 3:**
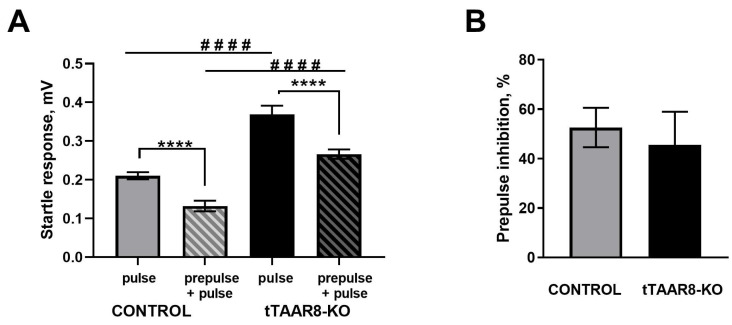
Evaluation of the effectiveness of sensorimotor gating in tTAAR8-KO and control mice in the prepulse inhibition test. (**A**) Amplitude of startle reflex in response to pulse and paired (prepulse and subsequent pulse) stimuli; (**B**) value of prepulse inhibition. **** *p* < 0.0001, #### *p* < 0.0001, Mann–Whitney test. All results are shown as mean ± SEM.

In both groups of animals (control and tTAAR8-KO), there was a significant decrease in the amplitude of startle response at the presentation of paired stimuli compared with the response to a single pulse stimulus (*p* < 0.0001) (Figure 3A). Both groups of animals demonstrated the presence of pre-impulse inhibition. A comparison of mutant and control mice by the parameter of the magnitude of pre-impulse inhibition did not reveal significant differences (Figure 3B).

Nevertheless, it was found that the amplitude of startle response of tTAAR8-KO animals was significantly greater than in control mice both in response to the presentation of single pulses (0.37 ± 0.02 mV and 0.21 ± 0.01 mV, *p* < 0.0001) and in response to paired stimuli (0.27 ± 0.05 and 0.13 ± 0.01 mV, *p* < 0.0001).

### 3.5. Power Spectra

**Figure 4:**
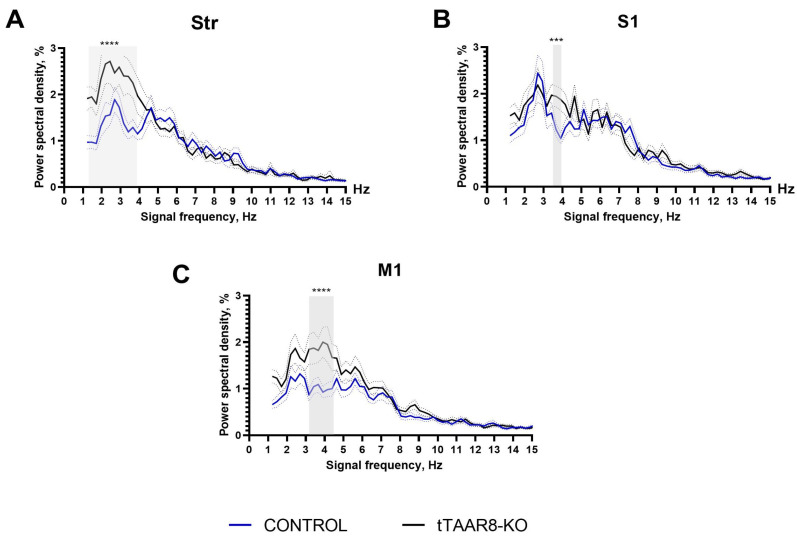
Electrophysiological power spectra in the brain regions of tTAAR8-KO vs. control mice. (**A**) Power spectral density of the local field potential in the striatum (Str). (**B**) Power spectral density of the primary sensory cortex (S1) electrocorticogram. (**C**) Power spectral density of the primary motor cortex (M1) electrocorticogram. Blue and black lines—the power spectra of tTAAR8-KO and control mice, respectively; dotted lines—standard error of the mean (SEM). *** *p* < 0.001, **** *p* < 0.0001, two-way ANOVA with the Sidak post hoc test.

The time–frequency characteristics of the ECoG of the primary motor cortex and striatum LFP were analyzed in free-moving, awake tTAAR8-KO and control group mice using the Fourier transform. The comparison was carried out using two-way ANOVA with the Sidak post hoc test in the frequency range of 1–15 Hz. A comparison by the group factor reveals significant differences in the striatum (F(1, 13909) = 33.57, *p* < 0.0001), sensory cortex (F(1, 13924) = 14.68, *p* = 0.0001), and motor cortex (F(1, 14042) = 72.34, *p* < 0.0001). According to the results of the post hoc analysis, an increase in signal power density was observed in the striatum (Str) of tTAAR8-KO compared with the control in the range of 1.2–3.9 Hz (*p* < 0.0001) (Figure 4A). Differences were also recorded in the primary sensory cortex (S1) (Figure 4B) and primary motor cortex (M1) (Figure 4C). In knockout animals, the signal power was significantly higher in the ranges of 3.7 Hz (*p* = 0.0005) and 3.9 Hz (*p* < 0.0001) for the sensory cortex and 3.2–4.4 Hz (*p* < 0.0001) for the motor cortex, respectively.

### 3.6. High-Performance Liquid Chromatography Study of Brain Monoamine Levels

**Figure 5:**
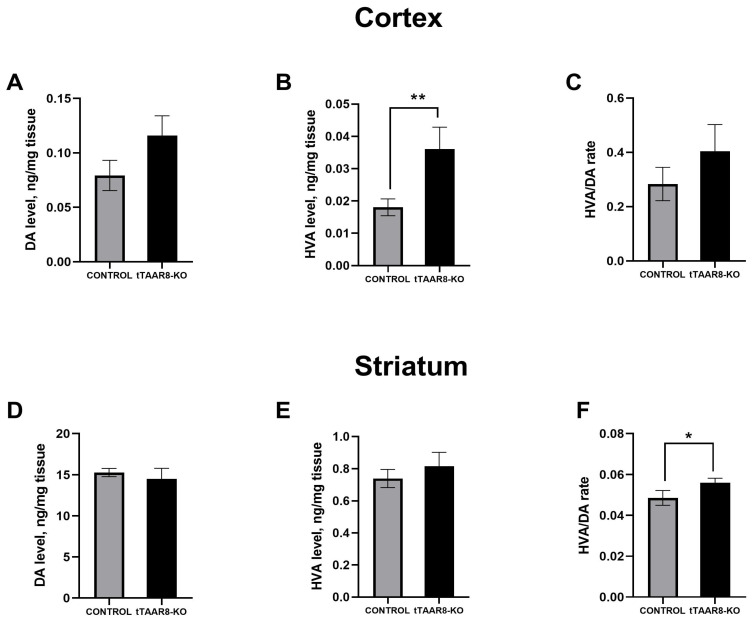
Alterations in DA metabolism in tTAAR8-KO mice. (**A**–**C**) Tissue level of DA, HVA, and HVA/DA rate in the frontal cortex of control and tTAAR8-KO mice. (**D**–**F**) Tissue level of DA, HVA, and HVA/DA rate in the striatum of control and tTAAR8-KO mice. DA—dopamine, HVA—homovanilinic acid. * *p* < 0.05; ** *p* < 0.01, Mann–Whitney test. All results are shown as mean ± SEM.

The quantitative content of NE, DA, DOPAC, HVA, 5-HT, and 5-HIAA in the cortex and striatum of mice was measured by the method of reverse-phase high-performance liquid chromatography. The HVA content in tTAAR8-KO mice in the frontal cortex was significantly higher compared to control mice (0.03156 ± 0.007 ng/mg of tissue, 0.0156 ± 0.0026 ng/mg of tissue, *p* < 0.01) (Figure 5B). The analysis of the levels of other monoamine neurotransmitters and their metabolites in the frontal cortex shows no significant differences.

In the striatum, there were no statistically significant differences in the level of most monoamines. However, it was found that tTAAR8-KO mice had a significantly increased HVA/DA ratio in the striatum compared to the control (0.056 ± 0.002, 0.049 ± 0.004, *p* < 0.05) (Figure 5F).

### 3.7. Tyrosine Hydroxylase Immunofluorescence

**Figure 6:**
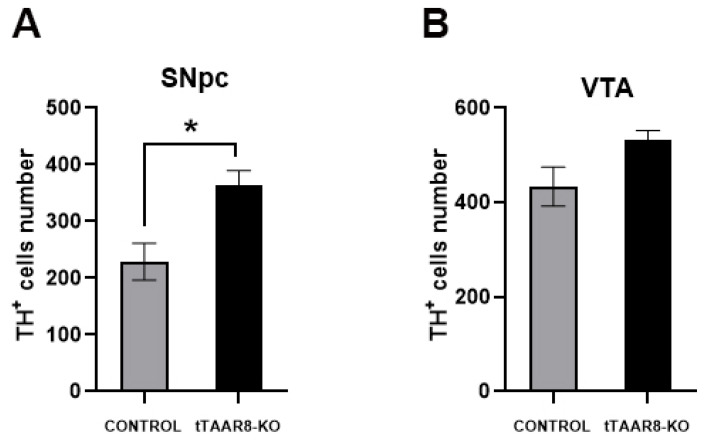
TH^+^ cell numbers in substantia nigra pars compacta (**A**) and ventral tegmental area (**B**) in tTAAR8-KO and control mice. TH—tyrosine hydroxylase, SNpc—substantia nigra pars compacta, VTA—ventral tegmental area. * *p* < 0.05, Mann–Whitney test. All results are shown as mean ± SEM.

We carried out TH fluorescent immunohistochemistry to determine whether the DA system changes in tTAAR8-KO mice. DA neurons in mice VTA and SNpc were identified by a positive fluorescent reaction on TH. The boundaries of each structure were determined according to the Allen Reference Atlas (http://atlas.brain-map.org/; accessed on 1 October 2023). We found that the number of SNpc TH^+^ neurons in tTAAR8-KO mice was higher than in controls (363.2 ± 26.23, 229 ± 32.62, *p* > 0.05) (Figure 6A). In VTA, we did not find significant differences in TH^+^ neuron numbers in tTAAR8-KO mice compared to control mice (532.8 ± 19.3, 433.5 ± 41.04, *p* = 0.06) (Figure 6B).

### 3.8. Adult Neurogenesis Analysis Immunofluorescence

**Figure 7:**
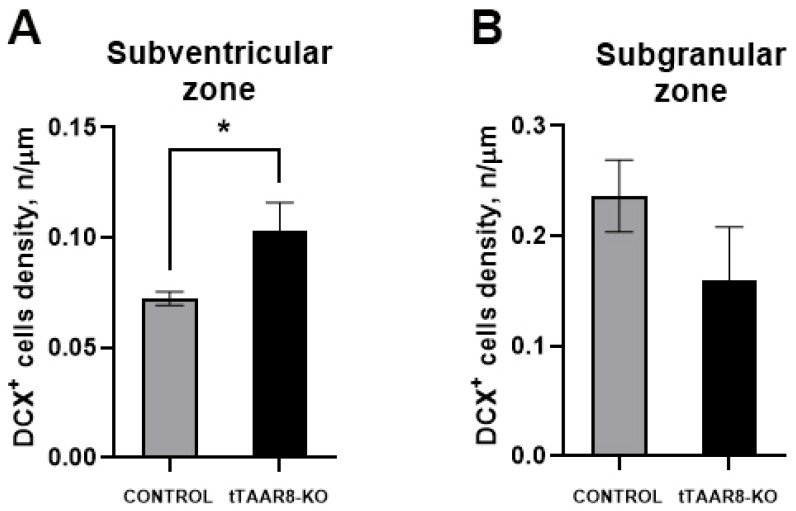
DCX^+^ neuroblast-like cell density in the subventricular zone of the lateral ventricle (**A**) and in the subgranular zone of the dentate gyrus (**B**) in tTAAR8-KO and control mice. DCX—doublecortin; SVZ—subventricular zone; SGZ—subgranular zone. * *p* < 0.05, Mann–Whitney test. All results are shown as mean ± SEM.

The immunofluorescence reaction against DCX allowed us to assess the neurogenesis level in mice lacking TAAR8. The analysis was carried out within two neurogenic niches of the mouse brain: SVZ and SGZ. We observed an increased density of neuroblast-like DCX^+^ cells in the SVZ of tTAAR8-KO mice (0.103 ± 0.01298, 0.07241 ± 0.003091, *p* < 0.05) (Figure 7A). We did not observe significant differences in the DCX^+^ neuron density in the SGZ of tTAAR8-KO mice compared to control mice (0.1601 ± 0.04794, 0.2364 ± 0.03268, *p* = 0.236) (Figure 7B).

## 4. Discussion

Gradually, evidence is accumulating that all TAAR family members are involved in brain functions and could be associated with mental and nervous disease pathogenesis [29,30]. TAAR1 has emerged as a promising therapeutic target, with agonists currently in clinical trials for schizophrenia [6,7,8,9]. The remaining TAARs, which have long been considered exclusively olfactory receptors, have also been shown to play a role in the mammalian brain [31]. According to transcriptomic data, *Taar2*, *Taar5*, *Taar6*, *Taar8*, and *Taar9* expression was found in the limbic and monoamine regions of the mammalian brain [28,32,33]. TAAR2-KO and TAAR5-KO mice display antidepressant-like phenotypes with altered dopaminergic and serotonergic neurotransmission and enhanced neurogenesis [11,12,26]. These findings motivate expanded investigation of TAARs in the brain, as well as in the aspect of mental and nervous diseases, along with TAAR1.

Previously, the mRNA of murine *Taar8* isoforms was found in several brain structures, namely, the amygdala, cerebellum, cortex, hippocampus, striatum, and olfactory bulb [1,27,28]. In rats, *Taar8a* expression was observed in the brain cortex, white matter, and cerebellum [34]. According to our data, *Taar8a* is also expressed in the dopaminergic areas (VTA, substantia nigra, and nucleus accumbens) and glutamatergic areas (prefrontal cortex and amygdala). Additionally, *Taar8a* mRNA was found in the hypothalamus, ventral hippocampus, and brainstem.

TAAR8 is one of the least studied among the other members of the TAAR family, and there is little information on its functions. The generation of tTAAR8-KO mice allowed us to fill this gap and study the role of TAAR8 in the mouse brain. To date, there are no similar studies performed on animals lacking *Taar8* isoforms. Here, we describe, for the first time, the effect of the knockout of corresponding genes on the brain state in mice.

tTAAR8-KO mice showed no distinct abnormalities in growth and development. In behavior testing, tTAAR8-KO mice showed no changes in basic locomotor and exploratory activity in the open field test. Anxiety levels measured in the elevated plus maze and light–dark transition tests were also unaltered.

In the novel object recognition test, the tTAAR8-KO mice explored novel objects more, both during the first day, when both objects were novel to them, and during the second day, when they had one novel object. As anxiety levels and exploratory activity were unchanged in the knockout mice, this could point to better short-term memory. The increased novel arm exploration in T-maze testing further supports superior short-term memory performance [35]. Rodents typically exhibit novelty preference upon re-exposure to testing apparatuses [35]. This behavior requires the retention of initial exposures to guide alternative selection. These paradigms assess working memory components, particularly short-term memory [35].

Further, we reveal that tTAAR8-KO mice demonstrated more depressive-like behavior under acute, unavoidable stress in the forced swimming test. tTAAR8-KO mice exhibited a first freezing response faster, and total immobilization time was higher than in controls. It is noteworthy that in previous studies, TAAR-KO animals, on the contrary, showed less depressive-like behavior: in the forced swimming test, TAAR2-KO had lower immobile time compared to the control, and TAAR5-KO mice in the learned helplessness test demonstrated learned helplessness to a lesser extent than the control [12,26].

The stress-induced hyperthermia test assessed the degree of change in body temperature under stress. At the first measurement, tTAAR8-KO mice showed higher body temperature compared to control mice. An increase in body temperature reflects the development of an autonomic response to stress since the introduction of a thermometer is itself a stressor [36]. However, tTAAR8-KO mice exhibited elevated temperatures upon first administration, showing that they have elevated base body temperature. The trace amine 3-iodothyronamine (3IT) is known to have a hypothermic effect. Several TAAR family members, TAAR1 and TAAR5, have proven to be 3IT targets [37,38]. TAAR8 was also expected to be activated by 3IT because of its high expression in murine hearts, but in vitro experiments failed to confirm this [27]. However, an indirect effect of this trace amine on the receptor cannot be ruled out [27].

Along with the battery of behavioral tests, we assessed sensorimotor filtering in the prepulse inhibition test. In both groups of animals, a significant decrease in the amplitude of the startle was observed when two sound stimuli were presented compared to the reaction to a single stimulus. It is known that the amplitude of startles depends on a large number of factors: line, sex, age, living conditions, stimulus volume, stress, etc. [39,40]. The amplitude can vary even within the same animal strain [41]. A comparison of tTAAR8-KO mice and control mice in terms of the magnitude of prepulse inhibition did not reveal significant differences. However, tTAAR8-KO mice showed a significantly more intense reaction to both single stimulus (pulse) and pairs of stimuli (prepulse + pulse). This may indicate the presence of disturbances in the system of involuntary attention to auditory stimuli in tTAAR8-KO.

As part of the electrophysiological studies, we recorded electrical activity from the dorsal striatum, primary somatosensory cortex, and primary motor cortex. The analysis of the frequency–time characteristics of ECoG and the LFP of tTAAR8-KO mice and the control group showed a significant difference between the two groups of animals in the signal power in the striatum in the range of slow delta waves (below 4 Hz). The greatest differences were also recorded in the primary sensory and primary motor cortex: in knockout animals, the signal power was significantly higher in the ranges of 3.7–3.9 Hz and 3.2–4.4 Hz, respectively. Increased signal power in the slow-wave range is observed in the slow-wave sleep phase, but recent studies have shown that slow-wave activity can also be observed in the wakeful state [42,43]. A similar state that arose during wakefulness was noted in mice in a state of pronounced hypodopaminergia [44]. Although we did not find a change in tissue DA levels, it was found that the content of HVA in the frontal cortex of tTAAR8-KO mice was higher than that in the control mice. Also, the HVA/DA ratio in the striatum was higher, pointing to increased levels of extracellular DA metabolism in the striatum of tTAAR8-KO mice compared to the control group.

tTAAR8-KO mice did not have alterations in 5-HT content, in contrast to TAAR5-KO mice, which are known to have reduced 5-HT levels in the striatum and hippocampus [12]. The absence of differences in 5-HT and metabolite content in tTAAR8-KO and control mice is consistent with the absence of differences in anxiety levels between these groups.

Interestingly, it was previously shown that TAAR2-KO and TAAR5-KO animals have increased numbers of DA neurons expressing TH in SNpc compared to wild-type mice [11,26]. In our work, we also analyzed the number of TH^+^ neurons. In the midbrain of mice, we found an elevated number of SNpc DA neurons in tTAAR8-KO mice compared to controls. However, TAAR5-KO and TAAR2-KO mice, together with an increased number of TH^+^ neurons, had an increased level of DA in tissues. In tTAAR8-KO, unchanged DA tissue levels with an increased number of DA neurons can be explained by increased DA metabolism, indicated by an increased turnover rate.

Since several TAARs (TAAR2 and TAAR5) have been shown to be associated with neurogenic processes, we were interested in whether the TAAR8 knockout affects the level of adult neurogenesis in mice. We studied the number of immature DCX^+^ neurons in the neurogenic niches of tTAAR8-KO and control mice. In contrast to TAAR2-KO and TAAR5-KO, which have an increased number of DCX^+^ immature neurons both in the SGZ and the SVZ [11,26], tTAAR8-KO mice have elevated neurogenesis only in the SVZ. The newborn neuron generation in adults could impact several physiological or behavioral reactions and be dependent on external stimuli [45,46]. Neurogenesis in the SGZ is important for hippocampal-dependent tasks, including learning, memory, anxiety regulation, novelty processing, and stress response [47,48], while neurogenesis in the SVZ is involved in the structural integrity of the olfactory bulb, olfactory memory, fear conditioning, and behavioral reactions to pheromones [49]. These alterations are consistent with the dopaminergic system state in tTAAR8-KO mice. Indeed, the data has demonstrated that DA is involved in neurogenesis not only during brain development but also postnatally [50,51].

The observed enhancement of adult neurogenesis in the SVZ of tTAAR8-KO mice might represent a compensatory mechanism aimed at maintaining olfactory function through the increased production of new olfactory neurons. This hypothesis aligns with data showing significant *taar* gene expansion in fish species, where these receptors play a crucial role in detecting water-soluble amines [52]. In terrestrial vertebrates, *Taars* likely underwent functional divergence, with some receptors (including *Taar8*) acquiring novel brain roles unrelated to direct chemosensation [53,54,55].

Notably, this pattern reflects a broader phylogenetic trend, where the *Taar* gene number positively correlates with the adult neurogenesis capacity across vertebrates. While teleost fish (e.g., *Danio rerio* with 112 *taar* genes) maintain robust constitutive neurogenesis supporting their chemosensory-dependent lifestyles, mammalian lineages show both reduced *Taar* repertoires (e.g., 6 functional in humans and 15 in mice) and restricted neurogenic zones, with cetaceans (0–2 *Taars*) representing the extreme case of *Taar* loss coinciding with minimal adult neurogenesis [56,57]. This correlation suggests that *Taars* may have evolutionarily co-opted roles in regulating neurogenic processes beyond their ancestral chemosensory functions.

The lack of selective ligands for TAAR8 significantly impedes functional investigations of this receptor. However, we could hypothesize that TAAR8 is likely responsive to tertiary amines—products of microbial amino acid decarboxylation [3]. Thus, similar to other TAARs, TAAR8 may function as a chemosensor, detecting metabolites produced during the bacterial decomposition of organic matter [58].

Furthermore, certain foods rich in trace amines (e.g., aged cheeses, fermented soy products, and cured meats) [59,60] could potentially activate TAAR8, as they contain structurally related biogenic amines. In particular, dietary tyramine and phenylethylamine have been shown to activate other TAAR subtypes [3,61], suggesting possible cross-reactivity. However, direct evidence linking specific food-derived compounds to TAAR8-mediated signaling remains scarce, highlighting the need for further pharmacological and nutritional studies in this field.

Most TAARs were previously considered exclusively olfactory, but emerging evidence suggests their involvement in neurotransmitter system regulation [12,26]. It appears that TAAR8 may function not only as an olfactory receptor but also participates in brain activity regulation. The most intriguing finding was the increased depressive-like behavior in tTAAR8-KO mice. Potentially, TAAR8 activation, similar to TAAR1 agonists, might alleviate depressive symptoms. Considering the results we obtained on tTAAR8-KO mice, it can be assumed that human TAAR8 could be involved in the modulation of the dopaminergic system, influencing brain activity and behavior.

Further studies on the mechanism of action, better understanding of which of mouse three isoforms corresponds to the single isoform in humans, the receptor expression pattern and its possible association with neuropsychiatric disorders, and more studies on single TAAR8-KO mice are needed to determine the role of TAAR8 in the regulation of neurotransmission, brain function, and behavior.

## Figures and Tables

**Table 1:** Oligonucleotides used in this study.

N	Oligonucleotide	Sequence	Purpose
1	sg RNA_taar8abc	5′-GCGGCCTCTAATACGACTCACTATAGGG GCCCCGGGTCATCCTGTACAGTTTTAGAGCTAGAAATAGCA-3′	Common sgRNA for three genes: *Taar8a*, *Taar8b*, *Taar8c*
2	TAAR8_F	5′-CTTCTAGCAGGACAGAAATCCC-3′	Common forward primer for PCR genotyping
3	TAAR8A_R	5′-AGAAAGCAACAGTATGCTTACATAC-3′	The reverse primer for PCR genotyping, specific for each gene
	TAAR8B_R	5′-CCAGGATAAACTAGAAGCTTAGAAAC-3′	
	TAAR8C_R	5′-TGAGATCACAGGGACCATCAG-3′	
4	Pr_taar8abc_R_sgABC	5′-ATCTCCAAAGTACCAGCAGCTC-3′	Common reverse primer for sequencing each gene: *Taar8a*, *Taar8b*, *Taar8c*

## Data Availability

Data is contained within the article or Supplementary Material.

## Supplementary Materials

The following supporting information can be downloaded at: https://www.mdpi.com/article/10.3390/biomedicines13061391/s1, Figure S1: Comparison of mouse gene sequences *Taar8a*, *Taar8b*, *Taar8c*; Figure S2: Sequence alignment of the nucleotide sequences comparing mouse taar8a, taar8b, and taar8c genes from the 3′-end of exon. The stop codon is highlighted in blue, and regions with 100% identity are marked in pink. Reverse primers were chosen from an adjacent region containing nucleotide variations, as shown in the frame.

## Abbreviations

The following abbreviations are used in this manuscript:

5-HIAA	5-hydroxyindoleacetic acid
5-HT	5-hydroxytryptamine, serotonin
DA	dopamine
DCX	doublecortin
DOPAC	3,4-dihydroxyphenylacetic acid
ECoG	electrocorticogram
GPCRs	G protein-coupled receptors
HVA	homovanillic acid
KO	knockout
LFP	local field potential
NE	norepinephrine
sgRNA	single guide RNA
SGZ	subgranular zone
SNpc	pars compacta of substantia nigra
SVZ	subventricular zone
TAARs	trace amine-associated receptors
TH	tyrosine hydroxylase
tTAAR8-KO	triple TAAR8abc–KO
VTA	ventral tegmental area
