# Supplementary material for: TAAR8 in the Brain: Implications for Dopaminergic Function, Neurogenesis, and Behavior
Source: Biomedicines. 2025 Jun 6;13(6):1391. doi: 10.3390/biomedicines13061391 (PMC12190788; doi:10.3390/biomedicines13061391)
Supplement: Supplementary file 1 [file biomedicines-13-01391-s001.zip › biomedicines-3626843-supplementary.pdf]

```
                 1                                                                    70
taar8a mm      (1) ATGACCAGCAACTTTTCCCAACCAGCCCTGCAGCTCTGCTATGAGAACACGAATGGATCCTGTATTAAAA
taar8b mm      (1) ATGACCAGCAACTTTTCCCAACCAGCCCTGCAGCTCTGCTATGAGAACACGAATGGATCCTGTATTAAAA
taar8c mm      (1) ATGACCAGCAACTTTTCCCAACCAGCCCTGCAGCTCTGCTATGAGAACACGAATGGATCCTGTATTAAAA

                   71                                                                  140
taar8a mm     (71) CTCCCTATTCTCCAGGGCCCCGGGTCATCCTGTACATGGTCTATGGCTTTGGGGCTGTGCTGGCAGTGTG
taar8b mm     (71) CTCCCTATTCTCCAGGGCCCCGGGTCATCCTGTACATGGTCTTTGGCTTTGGGGCTGTGCTGGCAGTGTG
taar8c mm     (71) CTCCCTATTCTCCAGGGCCCCGGGTCATCCTGTACATGGTCTATGGCTTTGGGGCTGTGCTGGCAGTGTG

                   141                                                                 210
taar8a mm    (141) TGGGAACCTCCTGGTGGTGATTTCAGTTCTCCATTTCAAGCAGCTGCACTCTCCAGCCAATTTTCTCATC
taar8b mm    (141) TGGGAACCTCCTGGTGGTGATTTCAGTTCTCCATTTCAAGCAGCTGCACTCTCCAGCCAATTTTCTCATC
taar8c mm    (141) TGGGAACCTCCTGGTGGTGATTTCAGTTCTCCATTTCAAGCAGCTGCACTCTCCAGCCAATTTTCTCATC

                   211                                                                 280
taar8a mm    (211) GCCTCTCTGGCCAGTGCTGACTTCTTGGTGGGCATCTCTGTGATGCCCTTCAGCATGGTCAGGTCCATTG
taar8b mm    (211) GCCTCTCTGGCCAGCGCTGACTTCTTGGTGGGCATCTCTGTGATGCCCTTCAGCATGGTCAGGTCCATTG
taar8c mm    (211) GCCTCTCTGGCCAGTGCTGACTTCTTGGTGGGCATCTCTGTGATGCCCTTCAGCATGGTCAGGTCCATTG

                   281                                                                 350
taar8a mm    (281) AGAGCTGCTGGTACTTTGGAGATGCATTTTGTAGCCTTCACAGTTGTTGTGATGTGGCTTTTTGTTACTC
taar8b mm    (281) AGAGCTGCTGGTACTTTGGAGATGCATTTTGTAGCCTTCACAGTTGCTGTGATGTGGCTTTTTGTTACTC
taar8c mm    (281) AGAGCTGCTGGTACTTTGGAGATGCATTTTGTAGCCTTCACAGTTGCTGTGATGTGGCTTTTTGTTACTC

                   351                                                                 420
taar8a mm    (351) TTCTGTCCTCCACCTCTGCTTCATCTCAGTGGACAGGTACATCGCTGTCACGGACCCTCTGGTCTATCCC
taar8b mm    (351) TTCTGCCCTCCACCTGTGCTTCATCTCAGTGGACAGGTACATTGCTGTCACAGACCCTCTGGTCTATCCC
taar8c mm    (351) TTCTGCCCTCCACCTGTGCTTCATCTCAGTGGACAGGTACATAGCTGTCACGGACCCTCTGGTCTATCCC

                   421                                                                 490
taar8a mm    (421) ACCAAGTTCACAGTGTCTGTGTCTGGAATTTGCATCAGCATCTCCTGGATTCTGCCCCTGGTGTACAGCA
taar8b mm    (421) ACCAAGTTCACAGTGTCTGTGTCTGGAATTTGCATCAGCATCTCCTGGATTCTGCCCCTGGTATACAGCA
taar8c mm    (421) ACCAAGTTCACAGTGTCTGTGTCTGGAATTTGCATCAGCATCTCCTGGATTCTGCCCCTGGTATACAGCA

                   491                                                                 560
taar8a mm    (491) GTGCAGTGTTTTACACAGGCATCAGTGCTAAGGGGATTGAAAGCTTAGTAAGTGCTCTAAATTGTGTAGG
taar8b mm    (491) GTGCAGTGTTCTACACAGGCATCAGTGCTAAGGGGATTGAAAGCTTAGTAAGTGCTCTGAATTGTGTAGG
taar8c mm    (491) GTGCAGTGTTCTACACAGGCATCAGTGCTAAGGGGATTGAAAGCTTAGTAAGTGCTCTGAATTGTGTAGG

                   561                                                                 630
taar8a mm    (561) GGGCTGCCAAATTGTTATCAATCAAGACTTTGTTTTGATAAGTTTTCTTCTATTCTTCATACCTACCCTT
taar8b mm    (561) GGGCTGCCAAATCGTTGTCAATCAAGACTGGGTTTTGATAGATTTTCTTCTATTTTTCATACCTACCCTT
taar8c mm    (561) GGGCTGCCAAGTTGTTGTCAATCAAGACTGGGTTTTGATAAGTTTTCTTTTATTCTTCATACCTACCGTT

                   631                                                                 700
taar8a mm    (631) GTTATGATCATTCTTTACAGCAAAATATTTTTGGTAGCCAAACAGCAAGCTGTAAAAATTGAAACTTCTG
taar8b mm    (631) GTTATGATCATTCTTTACAGCAAAATATTTTTGGTAGCCAAACAGCAAGCTGTAAAAATTGAAACTTCTG
taar8c mm    (631) GTTATGATCATTCTTTACAGCAAAATATTTTTGGTAGCCAAACAGCAAGCTGTAAAAATTGAAACTTCTG

                   701                                                                 770
taar8a mm    (701) TAAGTGGCAACAGAGGTGAATCATCCTCAGAGAGTCACAAAGCCAGAGTGGCCAAGAGAGAGAGGAAGGC
taar8b mm    (701) TAAGTGACAACAGAGGTGAATCATCCTCAGAGAGTCACAAAGCCAGAGTGGCCAAGAGAGAGAGGAAGGC
taar8c mm    (701) TAAGTGGCAACAGAGGTGAATCATCCTCAGAGAGTCACAAAGCCAGAGTGGCCAAGAGAGAGAGGAAGGC

                   771                                                                 840
taar8a mm    (771) TGCAAAAACCTTGGGGGTCACTGTGGTGGCTTTTATGGTCTCGTGGCTCCCATACACAATTGACGCATTG
taar8b mm    (771) TGCAAAAACCTTGGGGGTCACTGTGGTGGCTTTTATGGTCTCGTGGCTCCCATACACAATTGACTCATTG
taar8c mm    (771) TGCAAAAACCTTGGGGGTCACTGTGGTGGCTTTTATGGTCTCGTGGCTCCCATACACAATTGATGCATTG

                   841                                                                 910
taar8a mm    (841) GTTGATGCTTTCATGGGCTTCATCACTCCTGCCTATGTCTATGAAATCTGTTGCTGGGGTACCTATTATA
taar8b mm    (841) GTTGATGCTTTCGTGGGCTTCATTACTCCTGCCTATGTCTATGAAATCTGTTGCTGGAGTGCCTATTATA
taar8c mm    (841) GTTGATGCTTTCATGGGCTTCATCACTCCTGCCTATGTCTATGAAATCTGTTGCTGGAGTGCCTATTATA

                   911                                                                 980
taar8a mm    (911) ACTCAGCCATGAACCCTTTGATTTATGCTTTCTTTTTTCCTTGGTTTAAGAAAGCCATAAAGCTTATTTT
taar8b mm    (911) ACTCAGCCATGAACCCTTTGATTTATGCTTTCTTTTATCCTTGGTTTAGGAAAGCCATAAAGCTTATTTT
taar8c mm    (911) ACTCAGCCATGAACCCTTTGATTTATGCTTTCTTTTATCCTTGGTTTAGGAAAGCCATAAAGCTTATTTT

                   981                                                 1035
taar8a mm    (981) AAGTGGGGAAATTCTAAAGGGACATTCATCAACTGCAAATTTATTTTCAGAGTAA
taar8b mm    (981) AAGTGGGGAGATTCTAAAGAGTCATTCATCTACCATGAGTTTGTTTTCAGAGTAA
taar8c mm    (981) AAGTGGGAAAATTCTAAAGGGACATTCATCAACTACAAATTTATTTTCAGAGTAA
```

Figure S1. Comparison of mouse gene sequences *taar8a*, *taar8b*, *taar8c*.

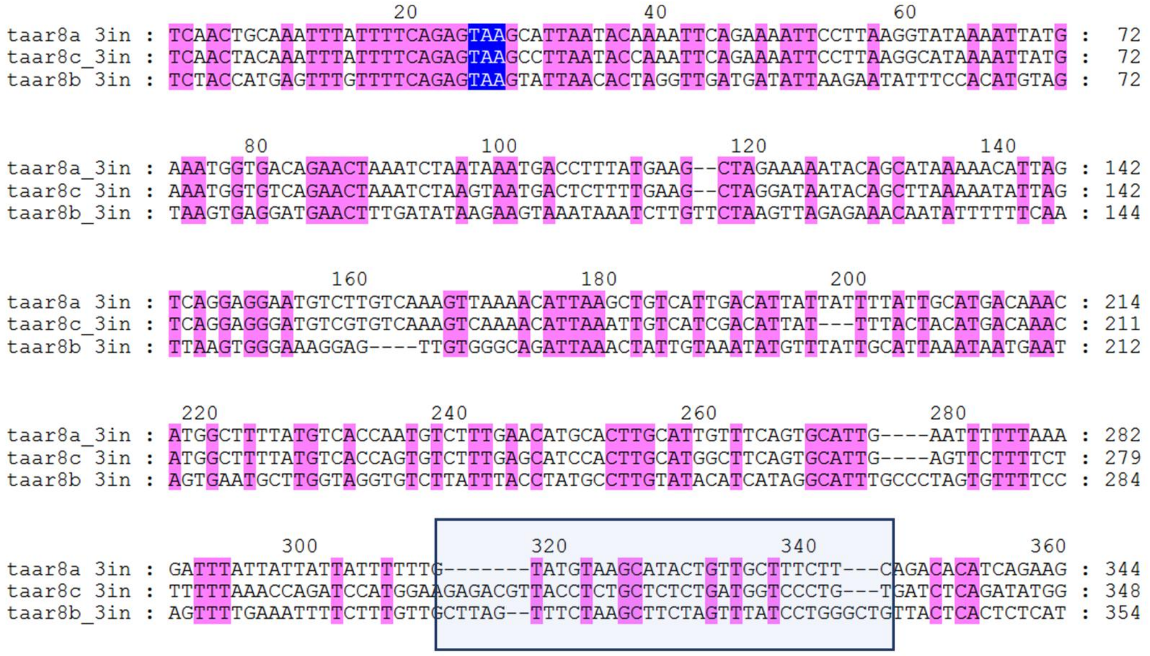


**Figure S2.** Sequence alignment of the nucleotide sequences comparing mouse *taar8a*, *taar8b*, and *taar8c* genes from the 3'-end of exon. The stop codon is highlighted in blue, and regions with 100% identity are marked in pink. Reverse primers were chosen from an adjacent region containing nucleotide variations, shown in frame.
